# Shiga Toxin–producing *Escherichia coli,* Idaho

**DOI:** 10.3201/eid1308.070189

**Published:** 2007-08

**Authors:** Vivian Marie Lockary, Richard Frederick Hudson, Christopher Lawrence Ball

**Affiliations:** * Idaho Bureau of Laboratories, Boise, Idaho, USA

**Keywords:** Escherichia coli, O157, immunoassay, hemolytic uremic syndrome, Idaho, Shiga toxin, letter

**To the Editor:** Data collected from expanded surveillance study suggest that more than half of Idaho Shiga toxin–producing *Escherichia coli* (STEC) illnesses are caused by non-O157 serotypes. Using data from a regional medical center whose stool culture protocol included Shiga toxin testing, we predicted Idaho’s STEC incidence to be significantly higher if non-O157 STEC were routinely detected by immunoassay. Recent findings suggest that the prediction was accurate in an expanded surveillance area.

Several studies have shown an increased incidence of non-O157 STEC infections in the United States. For example, a community hospital in Virginia detected non-O157 serotypes in 31% of patients with STEC from 1995–2002 (*1*). A 1998 Nebraska study that analyzed 30,000 diarrheal stool samples found that non-O157 and O157:H7 STEC were equally prevalent (*2*). Additionally, findings from a Connecticut study of laboratory-confirmed cases (*3*), STEC surveillance results from Montana (*4*), and a recent study from Michigan (*5*) indicate that non-O157 serotypes comprise a substantial percentage of STEC cases.

In other countries, nonculture-based methods are routinely used for STEC detection (*6*). However, *E. coli* O157:H7 culture methods remain the focus in the United Kingdom, Canada, and the United States (*6*). Reliance on culture methods can result in misleading interpretations of STEC prevalence. For example, 93% of STEC infections in Canada are reported to be *E. coli* O157:H7, yet a Manitoba 1992 study showed that when toxin assays were used, 35% of the recovered STEC isolates were non-O157 serotypes (*6*).

Analysis of reported non-O157 STEC cases in Idaho showed a similar trend. From 2002–2004, 66% of Idaho’s non-O157 cases originated in Health District 7, where >70% of stool cultures are screened by enzyme immunoassay (EIA) for Shiga toxin (Premier EHEC, Meridian Bioscience, Cincinnati, OH, USA). This rate was disproportionately higher than that of the remaining 6 health districts, which primarily use culture methods to screen for *E. coli* O157:H7. We hypothesized that this disproportion was due to differences in stool culture protocol. To test this premise, we conducted enhanced surveillance for 16 months in a “low” STEC incidence area, Health District 5. A total of 2,065 stools submitted for culture were screened for Shiga toxin by EIA. With this approach, reported non-O157 STEC incidence rose from <1 case/year/100,000 population to 11 cases/year/100,000 population. Additionally, 56% of recovered STEC isolates were non-O157 serotypes, mirroring the proportion of non-O157 detected in District 7. Notably, this appears to be the endemic rate for District 5 because no non-O157 STEC outbreaks or matching pulsed-field gel electrophoresis patterns were detected during the surveillance period. Although our study captured only a portion of stool cultures in Idaho, our findings demonstrated an increased prevalence of non-O157 STEC in the region when nonculture methods were used.

Two barriers cited for not routinely screening diarrheal stools for Shiga toxin are cost and perception of low non-O157 STEC incidence. While toxin testing is more expensive than culture testing, the potential effects of misdiagnosis may outweigh cost concerns. A study estimating the financial repercussions of *E. coli* O157 infections in the United States suggested that annual cost associated with this pathogen is $405 million, with the cost per case varying from $26 for those who do not seek medical care to $6.2 million for a patient with fatal hemolytic uremic syndrome (HUS) (*7*). Non-O157 STEC infections have been an important cause of HUS in many countries. For example, a 3-year prospective study in Germany and Austria reported that non-O157 serotypes comprised 90 (43%) of 207 STEC isolates from stools of 394 pediatric patients with HUS (*8*). Further, a 6-year Danish study of 343 registered STEC patients found that 76% of STEC and 48% of HUS cases were attributable to non-O157 serotypes (*9*). In the United States, continued reliance on O157 STEC culturing hinders our ability to determine the financial effects and the proportion of HUS cases attributable to non-O157 STEC.

Some evidence suggests that the testing focus may be changing in the United States. We used US Census Bureau population statistics to translate reported O157:H7 and non-O157 STEC cases for each state into incidence data. Despite widespread variation in STEC testing and incidence among states, there has been a significant statistical decline in the proportion of *E. coli* O157:H7 among total STEC cases every year since 2001 (Figure; p<0.001) (*10*). Consistent with this trend, the incidence of non-O157 STEC in the United States has increased (*10*). This may indicate that more laboratories are adopting Shiga toxin testing protocols, as we are advocating in Idaho. Our findings suggest that perceptions of low non-O157 STEC incidence in Idaho are probably artifactual and due to overemphasis on culture methods for O157 STEC. Our ongoing EIA-based surveillance highlights the need for continued investigation of the epidemiology of non-O157 STEC disease. We conclude that O157 STEC culturing has limited usefulness in areas like the Idaho health districts investigated, where non-O157 serotypes accounted for 55% of STEC illnesses. The true involvement of non-O157 in STEC disease will remain obscured as long as screening methods focus on traditional culture methods.

**Figure:**
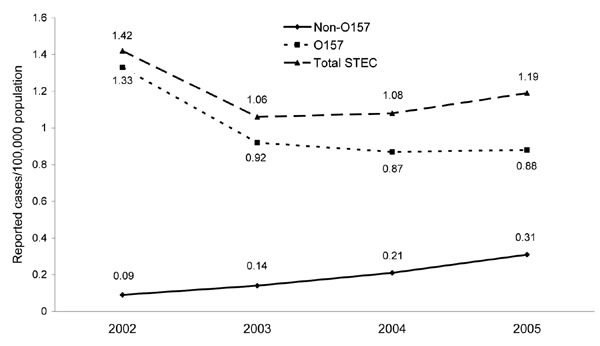
Shiga toxin–producing *Escherichia coli* (STEC) incidence trends, United States, 2002–2005.
